# The PI3K/AKT/mTOR pathway is a potential predictor of distinct invasive and migratory capacities in human ovarian cancer cell lines

**DOI:** 10.18632/oncotarget.4550

**Published:** 2015-07-14

**Authors:** Huimin Bai, Haixia Li, Weihua Li, Ting Gui, Jiaxin Yang, Dongyan Cao, Keng Shen

**Affiliations:** ^1^ Department of Obstetrics and Gynecology, Peking Union Medical College Hospital, Chinese Academy of Medical Sciences & Peking Union Medical College, Beijing China; ^2^ Department of Obstetrics and Gynecology, Beijing Chao-Yang Hospital, China Capital Medical University, Beijing China

**Keywords:** ovarian cancer, intratumoral heterogeneity, cell subclones, invasion and migration, RNA-Seq

## Abstract

**Objectives:**

To explore the genetic and molecular events that control subclones exhibiting distinct invasive/migratory capacities derived from human epithelial ovarian cancer (EOC) cell line A2780 and SKOV3.

**Methods:**

Single-cell subclones were isolated and established that were derived from the SKOV3 and A2780 cell lines through limiting dilution methodology. Transwell insert assays and MTT assays were performed to screen and identify the subclones exhibiting the highest and the lowest invasive/migratory capacities, and the selected subclones were renamed as A-H (A2780 high), A-L (A2780 low), S-H (SKOV3 high), and S-L (SKOV3 low). Their biological characteristics were evaluated. RNA-Seq was conducted on the targeted subclones.

**Results:**

Compared with their corresponding counterparts, A-H/S-H cells exhibited significantly higher invasive/migratory capacities (*P* < 0.001 and = 0.001, respectively). A-H/S-H cells displayed a clear reduction in doubling time (P = 0.004 and 0.001, respectively), and a significant increase in the percentage of cells in S phase (*P* = 0.004 and 0.022, respectively). Additionally, the apoptotic rates of A-H/S-H cells were significantly lower than those of A-L/S-L cells (*P* = 0.002 and 0.026, respectively). At both mRNA and protein levels, caspase-3 and caspase-7 expression were reduced but Bcl-2 expression was increased in A-H/S-H cells. The TrkB (anoikis-related) and Beclin1 (autophagy-related) levels were consistently high and low, respectively, in both A-H/S-H cells. Resistance to chemotherapy *in vitro* and higher capacities on tumor formation *in vivo* was presented in both A-H/S-H cells. PI3K/AKT/mTOR pathway components, PIK3CA, PIK3CD, AKT3, ECM1, GPCR, mTOR and PRKCB were increased but that the Nur77 and PTEN were decreased in A-H/S-H cells, identified by RNA-Seq and consistently confirmed by RT-PCR and Western blot analyses.

**Conclusions:**

Heterogeneous cell subpopulations exhibiting distinct invasive and migratory capacities co-exist within the SKOV3 and A2780 cell lines. PI3K/AKT/mTOR pathway activation is associated with higher invasive and migratory capacities in subpopulations of human ovarian cancer cell lines. Inhibiting this pathway may be useful for the chemoprevention or treatment of EOC.

## INTRODUCTION

Epithelial ovarian cancer (EOC) is the most lethal gynecologic malignancy [1]. Intratumoral heterogeneity (ITH) has been attributed to treatment failure in many human malignancies [2–4]. The divergence of cell subpopulations in tumors confers a heterogeneous genomic landscape with implications on clinical prognosis, including the acquisition of metastatic potential and chemotherapy resistance [5]. RNA-Seq provides unprecedented possibilities for genomic characterization; this technique has significantly advanced our understanding of genomic organization, including allele-specific expression, novel transcripts, and unique isoforms [6]. RNA-Seq technology may be helpful in identifying pathways and molecules that may be relevant for therapeutic intervention in EOC.

Heterogeneity is a common phenomenon of parental tumor cell lines, and highly metastatic tumor cell variants pre-exist in parental tumor cell populations [7–9]. SKOV3 cells, which were isolated from ascites of a patient with ovarian adenocarcinoma, are resistant to several cytotoxic drugs, including cisplatin [10]. In contrast, A2780 cells, which were derived from an adenocarcinoma patient prior to treatment, are sensitive to cisplatin [11]. Both of these two cell lines are popular research models for human EOC. Based on previous studies, we isolated and established two pairs of cell subclones exhibiting distinct invasive/migratory capacities that were derived from the SKOV3 and A2780 cell lines. Each pair of subclones with the same hereditary background served as a model of intra-tumor heterogeneity, and consistent findings in these two EOC cell lines provided additional convincing data. The present study aimed to investigate the disparities in gene expression between the targeted subclones using RNA-Seq. Determining the genetic and molecular events that control the distinct invasive/migratory capacities of these subclones will facilitate our understanding of EOC treatment failure and will lead to the development of more effective therapeutics.

## MATERIALS AND METHODS

### Materials and cell culture

The principal materials used in this study are shown in supplementary (S)-Table 1. Cells were cultured in RPMI-1640 supplemented with 10% fetal bovine serum (FBS) and antibiotics (100 U/ml penicillin and 100 μg/ml streptomycin) at 37°C in a humidified atmosphere in a 5% CO_2_ incubator.

### Isolation and amplification of single-cell subclones

Limiting dilution methodology was performed as described by Chen et al. [12]. A2780 and SKOV3 cells were collected in the logarithmic growth phase, adjusted to a density of 10 cells/ml, and then seeded into 96-well plates (0.1 ml/well). Eight 96-well plates were seeded for each cell line. The 96-well plates were observed under a microscope, and wells containing a single cell were marked. Single-cell subclones proliferated to a certain density after culturing for 2–3 weeks; then, the subclones were transferred to 48-well plates. The subclones were successively transferred to 24-, 12- and 6-well plates and, finally, to culture dishes. All of the subclones were amplified, and back-up subclones were stored in liquid nitrogen.

### Matrigel invasion assay and cell migration assay

Transwell insert assays and 3-(4, 5-dimethyl-2-thiazolyl)-2, 5-diphenyl-2-H-tetrazolium bromide (MTT) assays were performed to assess and compare the invasive/migratory capacities of the subclones. Polyvinylpyrrolidone-free polycarbonate (PVP-free) filters with a pore size of 8.0 μm, which were either uncoated (migration assay) or coated (invasion assay) with 50 μl of Matrigel diluted 1:3 in serum-free RPMI-1640, were placed in Transwell chambers. Cells (0.1 × 10^6^) in the logarithmic growth phase were suspended in 200 μl of serum-free RPMI-1640 and seeded in the upper chambers of Matrigel-coated Transwell plates. RPMI-1640 containing 20% FBS (600 μl) was added to the lower chamber as a chemotactic factor. Each subclone was seeded in triplicate. All of the Transwell chambers were then incubated at 37°C for 24 h. Non-invading cells that remained on the upper surface of the filter were carefully removed with a swab. The Transwell chambers were then immersed in 500 μl of culture medium containing 0.5 mg/ml MTT and were incubated in the dark at 37°C for 4 h. Then, the liquid was discarded, 500 μl of dimethyl sulfoxide (DMSO) was added, and the cells were incubated at 37°C for 10 min to fully dissolve the formazan crystals. The absorbance at 490 nm was measured using a microplate reader. Based on their absorbance values, the subclones were classified into groups exhibiting high, moderate or low invasive/migratory capacity. Groups exhibiting high or low invasive/migratory capacity were retained and re-subjected to the above test. The subclones exhibiting the highest or the lowest invasive/migratory capacity were identified based on repeated comparisons and were renamed as A-H (A2780 high), A-L (A2780 low), S-H (SKOV3 high), and S-L (SKOV3 low). To investigate the stability of the invasive/migratory potential, populations of the A-H, A-L, S-H, and S-L cells were serially passaged to approximately the thirtieth generation, and the variation in the invasive/migratory capacities of the targeted subclones was evaluated.

The selected A-H, A-L, S-H, and S-L subclones were ultimately evaluated and analyzed using the following experimental procedures.

### MTT assays and growth curves

MTT assays were performed in 96-well plates. Cells (2 × 10^3^) were seeded in each well. Each subclone was seeded in six wells of each 96-well plate (eight 96-well plates in total). All of the 96-well plates were incubated at 37°C. One 96-well plate was removed from the incubator each day. Twenty microliters of MTT (5 mg/ml MTT in phosphate-buffered saline, PBS) was added to each well, and the plates were incubated in the dark at 37°C for 4 h. The liquid was then discarded, and 200 μl of DMSO was added to dissolve the formazan crystals. The optical density (OD) was measured at 490 nm using an enzyme-linked immunosorbent assay (ELISA) plate reader. The OD values for each subclone were recorded each day. Growth curves were plotted, and the doubling time was calculated.

### Cell cycle distribution assays

The four subclones were collected in the logarithmic growth phase and counted. A minimum of (5–10) × 10^5^ cells were washed twice with PBS, resuspended in 1 ml of cold ethanol (−20°C), and then incubated at −20°C for at least 30 min. The cells were centrifuged, and the supernatant was discarded. After the cells were washed twice with PBS, they were resuspended and stained with 500 μl of PBS solution containing 50 μg/ml propidium iodide (PI), 100 μg/ml RNase A, and 0.2% Triton X-100. The samples were incubated in the dark at 4°C for 30 min and then analyzed via flow cytometry. Approximately 20,000 cells were examined per sample. This assay was performed in triplicate.

### Apoptotic rates of A-H, A-L, S-H and S-L

The apoptotic rates were measured using an Annexin V-FITC/PI Apoptosis Detection Kit. Cells were collected in the logarithmic growth phase, washed twice with cold PBS, and adjusted to a density of 1 × 10^6^/ml in 1× binding buffer. The cell suspension (100 μl) was placed in a Falcon tube and was sequentially incubated in annexin V-FITC (5 μl) followed by PI (5 μl) at 20°C–25°C in the dark for 15 min. Then, 1× binding buffer (400 μl) was added, and the samples were analyzed by flow cytometry within one hour.

### RNA isolation and real-time polymerase chain reaction (RT-PCR)

The mRNA and protein levels of caspase-3, caspase-7, and Bcl-2 (apoptosis-related factors), TrkB (anoikis-related factor), and Beclin1 (autophagy-related factor) were detected. Total RNA was extracted using TRIzol reagent according to the manufacturer's instructions, and cDNA was then synthesized using a QuantScript Reverse Transcription Kit. All of the primers are listed in S-Table 2. Real-time PCR was performed using an Applied Biosystems 7500 Real-Time PCR System and a SuperReal PreMix Kit. The results were analyzed using the 2^(−ΔΔCT)^ comparative method [13]. Each sample was tested in triplicate.

### Western blot

Cells were lysed in cold radioimmunoprecipitation assay (RIPA) buffer containing freshly added 0.01% protease inhibitor for 30 min. After the lysed cells were incubated on ice for 10 min, they were centrifuged at 12000 × *g* for 10 min at 4°C, and the supernatants were collected. Approximately 50 μg of total protein was denatured for 10 min at 95°C, separated on a 10–15% SDS-polyacrylamide gel, and transferred to a nitrocellulose membrane. The membrane was blocked with 5% non-fat milk in Tris-buffered saline containing 0.1% Tween-20 (TBST) for 2 h at room temperature and then incubated in primary antibodies overnight at 4°C. The primary antibodies were detected by incubating the membranes in horseradish peroxidase-conjugated secondary antibodies for 2 h at room temperature, and the signals were visualized using a SuperEnhanced Chemiluminescence Detection Kit. Details of the primary and secondary antibodies are presented in S-Table 3. Each assay was performed in triplicate.

### Drug cytotoxicity assays

Cells were seeded at a density of 8,000 cells per well in 96-well plates. After the cells were incubated for 24 h, they were treated with different concentrations of cisplatin and taxol. MTT assays were performed after a 48-h incubation. Dose-response curves were then generated, and the 50% inhibitory concentration (IC50) values were calculated. Each assay was performed in triplicate.

### Ovarian tumor xenograft model in nude mice

Female BALB/c nude mice (4–5 weeks-old) were maintained in micro-isolator cages. Forty mice were randomly divided into 4 groups of 10 mice each. Xenografts were established by subcutaneously injecting the mice with 1 × 10^6^ cells. The tumor volumes were estimated using the formula (width)^2^ × length/2. The mice were euthanized after 30 days of observation, and the tumors were harvested. All procedures were reviewed and approved by the Institutional Animal Care and Use Committee of Peking Union Medical College Hospital.

### Transcriptome expression profiling

First, the library for Illumina sequencing was prepared. Briefly, 150 μg of total RNA was extracted from each sample using TRIzol reagent, according to the manufacturer's instructions. The quality of the extracted RNA was evaluated via 1.5% agarose gel electrophoresis and PI taining. The extracted RNA was then dissolved in RNase-free water and purified using an RNeasy Mini RNA Purification Kit. Illumina-compatible libraries were constructed using a TruSeq RNA library preparation kit. Briefly, mRNA was purified from the extracted RNA using oligo-dT magnetic beads and was fragmented. First-strand cDNA was synthesized using random hexamers and reverse transcriptase. Second-strand cDNA was synthesized using high quality deoxyribo nucleotide triphos phates (dNTPs), ribonuclease H (RNase H), and DNA polymerase. Double-stranded (ds) cDNA was synthesized via PCR amplification using sequencing adaptors. Fragments of 250–500 bp were selected, subjected to 2.5% agarose gel electrophoresis, excised from the gels, and purified using a Qiagen Gel Extraction Kit. The final library was sequenced on a HiSeq™ 2000 platform (Illumina).

Second, the obtained raw reads were analyzed by trimming, filtering, and the sequences aligning to the human genome (hg19) using TopHat (version 1.3.3) [14]; Cufflinks software (version 1.2.1) [15] was used to identify the differentially expressed genes. The transcript counts were calculated to measure gene expression, and the relative transcript abundance was assessed as fragments per kilobase of exon per million fragments mapped (FPKM) using Cufflinks software (version 1.2.1) [15]. Transcripts displaying a *P*-value < 0.05 and a-fold change in expression > 2.0 were considered to be significantly differentially expressed.

RT-PCR and Western blot analyses were performed to validate genes that exhibited significant differential expression in the RNA-Seq data for the targeted subclones. The primers and antibodies used are shown in S-Table 2 and S-Table 3, respectively.

### Statistical analysis

All statistical analyses were performed using SAS^®^ Version 9.2 (SAS Institute, Cary, NC). All tests were 2-sided, and *P* < 0.05 was considered to be statistically significant. The invasive/migratory capacities, cell doubling time, and tumor volumes were compared using two- tailed independent- sample *t* tests. Chi-square tests or Fisher's exact tests were performed to compare the apoptotic rates and cell cycle distribution.

## RESULTS

### Establishment of subclones exhibiting distinct invasive/migratory capacity

**Figure 1:**
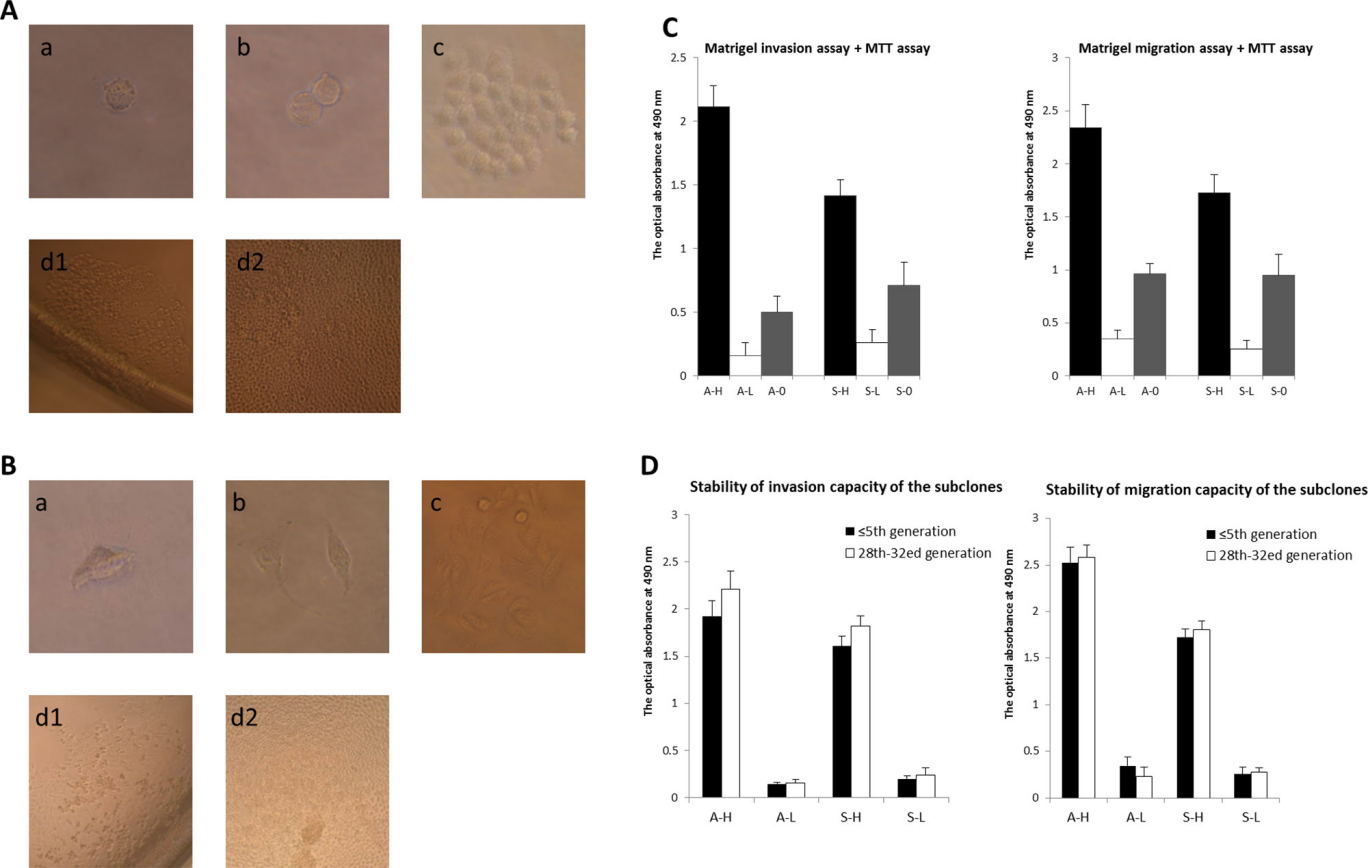
Isolation and establishment of subclones exhibiting distinct invasive/migratory capacity **A–B.** Isolation and amplification of single-cell subclones derived from human epithelial ovarian cancer cell lines A2780 (A) and SKOV3 (B) using limited dilution methodology. Single adherent A2780/SKOV3 cells were observed as early as 4–5 h after isolation and inoculation (a). Single cells began to divide, and 2–3 cells were observed in some wells of the plates 24 h after inoculation (b). The cell number continued to increase 4 days after inoculation (c). These single-cell subclones significantly differed in cell number of and subclone size ten days after inoculation (d1and d2). **C.** In the Matrigel invasive/migratory assay, subclone A6 (A-H) demonstrated the highest invasive/migratory activity, and subclone A41 (A-L) displayed the weakest ability to invade and migrate through the membrane (*P* < 0.001 and = 0.001, respectively). The invasive/migratory abilities of A-H and A-L both were significantly different from those of their parent cell line (A-H vs. A2780: *P* = 0.001 and 0.002, respectively; A-L vs. A2780: *P* = 0.044 and 0.001, respectively). Similarly, S15 (S-H) and S4 (S-L) exhibited the most distinct invasive/migratory abilities (*P* = 0.001 and < 0.001, respectively) among all the SKOV3 subclones. The invasive/migratory capacities of both S-H and S-L were significantly different from those of their parent cell line (S-H vs. SKOV3: *P* = 0.019 and 0.018, respectively; S-H vs. SKOV3: *P* = 0.037 and 0.018, respectively). **D.** The respective invasive/migratory capacities of A-H, A-L, S-H, and S-L were not significantly altered by serial passaging to approximately the thirtieth generation. After passaging to approximately 30 generations, the difference in the invasive/migratory capacities between A-H/S-H and A-L/S-L remained significant (invasion: *P* = 0.015 and < 0.001, respectively; migration: *P* < 0.001 and < 0.001, respectively).

Single adherent A2780/SKOV3 cells were observed as early as 4–5 h after isolation and inoculation (Fig. 1A and 1B). Single cells began to divide, and 2–3 cells were observed in some wells of the plates 24 h after inoculation. The cell number continued to increase 4 days after inoculation. These single-cell subclones significantly differed in cell number and subclone size ten days after inoculation.

For the A2780 cell line, single adherent cells were observed in 258 wells of the eight 96-well plates. Of these 258 single-cell clones, 166 died and disappeared, and 16 were eliminated due to bacterial contamination. Of the remaining 76 single-cell clones, 18 died and disappeared, and 13 were subsequently eliminated due to bacterial contamination during the transfer process from 96-well plates to 48-, 24-, 12-, and 6- well plates and, ultimately, to culture dishes. For the A2780 subclones, the contamination rate was 11.2%, and the mortality rate was 80.3%. Forty-five (17.4%) cell subclones were obtained and named A1–A45. For the SKOV3 cell line, single adherent cells were observed in 288 wells of the eight 96-well plates. Of these 288 single-cell clones, 148 died and disappeared, and 20 were eliminated due to bacterial contamination. Forty (19.2%) single-cell subclones were obtained and named S1–S40.

In the Matrigel invasive/migratory assay, subclone A6 (A-H) demonstrated the highest invasive/migratory activities, and subclone A41 (A-L) displayed the weakest ability to invade and migrate through the membrane (migration: 2.118 ± 0.161 vs. 0.162 ± 0.099, *P* < 0.001; invasion: 2.340 ± 0.322 vs. 0.350 ± 0.082, *P* = 0.001; Fig. 1C). The invasive/migratory abilities of A-H and A-L both were significantly different from those of their parental cell line (A-H vs. parental: *P* = 0.001/0.002; A-L vs. parental: *P* = 0.044/0.001). Similarly, S15 (S-H) and S4 (S-L) exhibited most distinct invasive/migratory abilities (invasion: 1.416 ± 0.127 vs. 0.261 ± 0.106, *P* = 0.001; migration: 1.726 ± 0.168 vs. 0.254 ± 0.082, *P* < 0.001) among all of the SKOV3 subclones. The invasive/migratory capacities of both S-H and S-L were significantly different from those of their parental cell line (S-H vs. parental: *P* = 0.019/0.018; S-L vs. parental: *P* = 0.037/0.018). The respective invasive/migratory capacities of A-H, A-L, S-H, and S-L were not significantly altered by serial passaging to approximately the thirtieth generation (Fig. 1D). After passaging to approximately 30 generations, the difference in invasive/migratory capacities between A-H/S-H and A-L/S-L remained significant (invasion: *P* = 0.015/*P* < 0.001; migration: *P* < 0.001/*P* < 0.001). These results indicated that the selected subclones had relatively stable biological traits. However, the invasive/migratory capacities of these subclones were slightly increased following passaging for thirty generations, with the exception of the migratory capacity of A-L, which decreased slightly, possibly due to experimental error. Consequently, subclones in similar generations were used, unnecessary passaging was avoided, and subclones in no greater than the 20^th^ generation were used in the further comparative analyses.

### Heterogeneous biological functions between A-H/S-H and A-L/S-L

**Figure 2:**
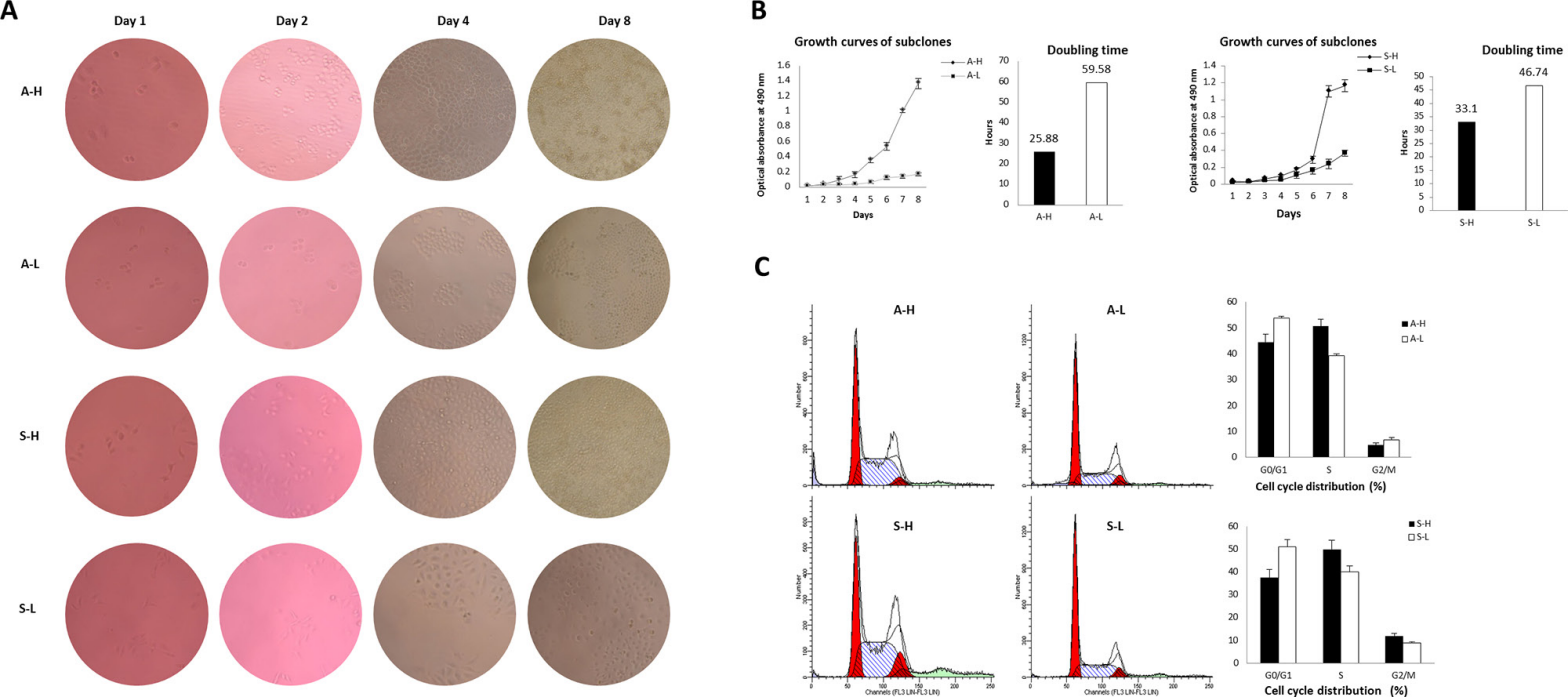
Distinct proliferative activities of A-H/A-L and S-H/S-L **A–B.** The MTT assay growth curves showed that A-H/S-H cells proliferated faster than A-L/S-L cells, based on a clear reduction in doubling time (*P* = 0.004 and 0.001, respectively). **C.** Cell cycle analysis indicated that the A-H/S-H subclones displayed a significantly increased in the percentage of cells in S phase (*P* = 0.004 and 0.022, respectively) and a concomitant decreased in the percentage of cells in G0/G1 phase (*P* = 0.012 and 0.007, respectively), suggesting that enhanced proliferation of the A-H/S-H cells might be due to the activation of DNA synthesis.

The MTT assay growth curves showed that A-H/S-H cells proliferated faster than A-L/S-L cells, based on a clear reduction in the doubling time (A: 25.88 ± 2.29 h vs. 59.58 ± 4.96 h, *P* = 0.004; S: 33.10 ± 1.19 h vs. 46.74 ± 2.31 h, *P* = 0.001; Fig. 2A, 2B). Cell cycle analysis indicated that the A-H and S-H subclones displayed a significant increase in the percentage of cells in S phase (A: 50.67 ± 2.78% vs. 39.31 ± 1.73%, *P* = 0.004; S: 49.91 ± 3.96% vs. 39.96 ± 2.62%, *P* = 0.022) and a concomitant decrease in the percentage of cells in G0/G1 phase (A: 44.60 ± 3.10% vs. 53.87 ± 1.94%, *P* = 0.012; S: 37.61 ± 3.55% vs. 51.18 ± 3.03%, *P* = 0.007), suggesting that the enhanced proliferation of A-H/S-H cells might be due to the activation of DNA synthesis (Fig. 2C).

**Figure 3:**
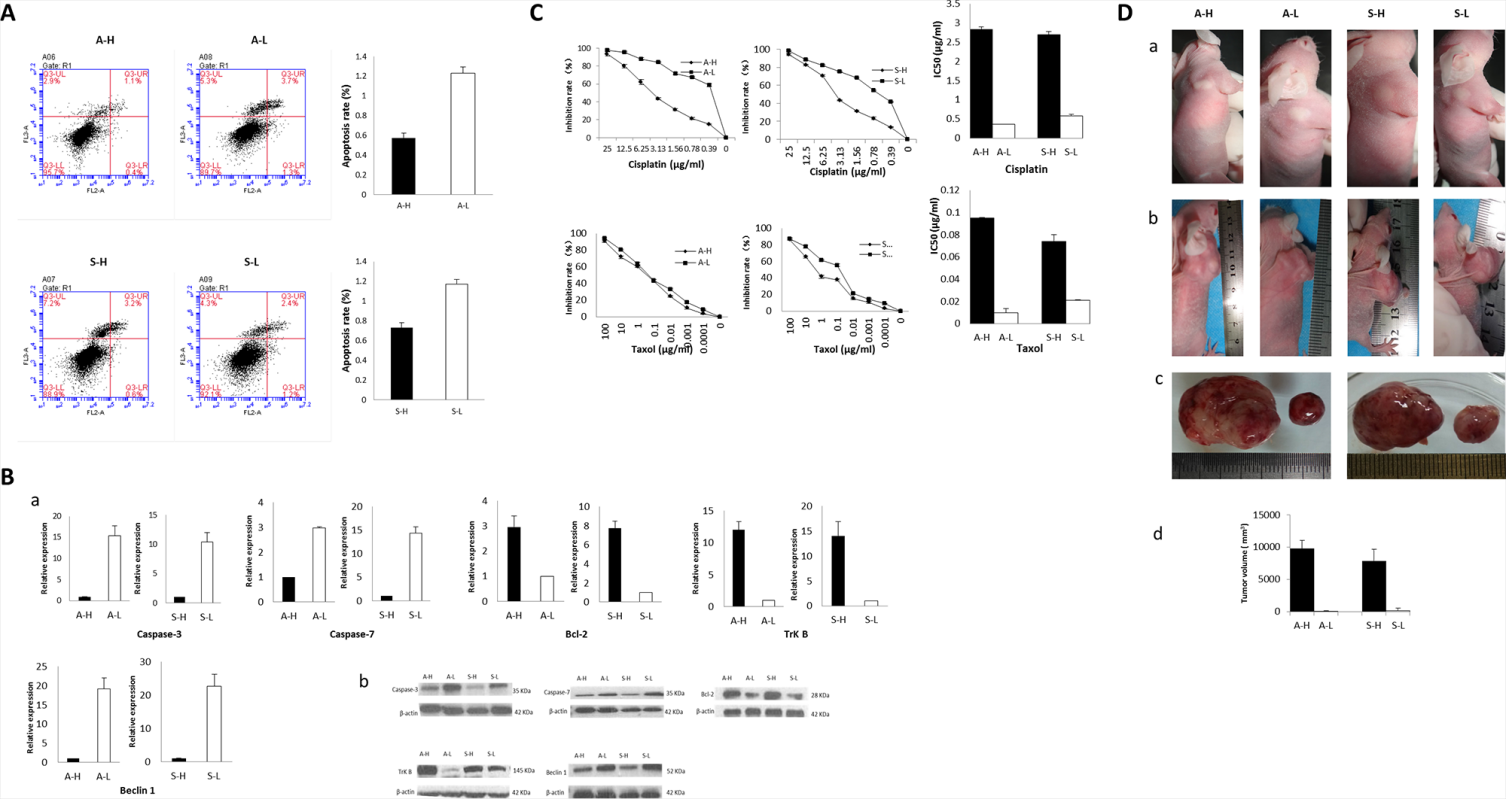
Heterogeneous biological activities of A-H/A-L and S-H/S-L **A–B.** The apoptotic rates of A-H/S-H cell were significantly lower than those of A-L/S-L cells (*P* = 0.002 and 0.026, respectively; A). At both the mRNA (a) and protein levels (b), caspase-3 and caspase-7 expression was reduced, but Bcl-2 expression was increased in A-H/S-H cells (B) Additionally, the TrkB and Beclin1 levels were consistently high and low, respectively, in both A-H and S-H cells. Compared with their corresponding counterparts, the A-H and S-H cells exhibited significantly greater proliferative, anti-apoptotic and anti-anoikis activities. Autophagic activity was suppressed in the A-H and S-H subclones. **C.** Cell viability after cisplatin or taxol treatment was significantly inhibited in A-L/S-L cells compared with A-H/S-H cells, as demonstrated by drug cytotoxicity assays. The sublones showing higher invasive/migratory capabilities (A-H/S-H) displayed markedly higher cisplatin IC50 values (*P* < 0.001 and < 0.0001, respectively). Similarly, the respective taxol IC50 values of A-H and S-H cells respectively were significantly higher than those of A-L cells and S-L cells (*P* = 0.002 and 0.002, respectively). The sublones exhibiting higher invasive/migratory capacities were more resistant to chemotherapy. **D.** Effect of heterogeneous invasive/migratory capacities for tumor formation *in vivo*. (a): After subcutaneous injection, A-H/S-H tumors were observed much earlier than A-L/S-L tumors (5th day vs. 19th day; 7th day vs. 20th day). (b-d): On the study end date (30 days after inoculation), the tumor formation rates of both the A-H/S-H cells were 100%, and these were significantly higher than those of the A-L and S-L cells, respectively (both 20%, *P* = 0.008) (b). Additionally, the A-H/S-H tumors were significantly larger than the A-L/S-L tumors (A: 2.34 ± 0.23 cm vs. 0.17 ± 0.33 cm, *P* < 0.001; S: 2.04 ± 0.61cm vs. 0.12 ± 0.27cm, *P* < 0.001). The A-H/S-H cells exhibited higher capacities for *in vivo* tumor formation.

The apoptotic rates of A-H/S-H cells were significantly lower than those of A-L/S-L cells (A: 0.57 ± 0.05% vs. 1.23 ± 0.06%, *P* = 0.002; S: 0.73 ± 0.05% vs. 1.17 ± 0.05%, *P* = 0.026; Fig. 3A). At both the mRNA and protein levels, caspase-3 and caspase-7 expression was reduced but Bcl-2 expression was increased in A-H/S-H cells (Fig. 3B). Additionally, the TrkB and Beclin1 levels were consistently high and low, respectively, in both A-H and S-H cells. Compared with their corresponding counterparts, the A-H and S-H cells exhibited significantly greater proliferative, anti-apoptotic and anti-anoikis activities. Moreover, autophagic activity was suppressed in the A-H and S-H subclones.

Cell viability after cisplatin or taxol treatment was significantly inhibited in A-L/S-L cells compared with A-H/S-H cells, as demonstrated by drug cytotoxicity assays (Fig. 3C). The subclones showing higher invasive/migratory capacities (A-H/S-H) displayed markedly higher cisplatin IC_50_ values (invasion: 2.83 ± 0.07 μg/ml vs. 0.37 ± 0.002 μg/ml, *P* < 0.001; migration: 2.70 ± 0.08 μg/ml vs. 0.58 ± 0.05 μg/ml, *P* < 0.001). Similarly, the respective taxol IC_50_ values of A-H and S-H cells were 0.095 ± 0.004 μg/ml and 0.074 ± 0.006 μg/ml, which were significantly higher than those of A-L cells (0.010 ± 0.004 μg/ml, *P* = 0.002) and S-L cells (0.021 ± 0.001 μg/ml, *P* = 0.002). The subclones exhibiting higher invasive/migratory capacities were more resistant to chemotherapy

### Effect of heterogeneous invasive/migratory capacities on tumor formation *in vivo*

After subcutaneous injection, A-H/S-H tumors were observed much earlier than A-L/S-L tumors (5^th^ day vs. 19^th^ day; 7^th^ day vs. 20^th^ day; Fig. 3D). On the study end date (30 days after inoculation), the tumor formation rates of both the A-H and S-H cells were 100%, and these rates were significantly higher than those of the A-L and S-L cells, respectively (both 20%; *P* = 0.008). Additionally, the A-H/S-H tumors were significantly larger than the A-L/S-L tumors (A: 9.8 ± 1.2 cm^3^ vs. 0.05 ± 0.1 cm^3^, *P* < 0.001; S: 7.9 ± 1.8 cm^3^ vs. 0.2 ± 0.4 cm^3^, *P* < 0.001). The A-H/S-H cells exhibited higher capacities for *in vivo* tumor formation.

### Transcriptome expression profiling according to the heterogeneous invasive/migratory capacities of human ovarian cancer cell lines

**Figure 4:**
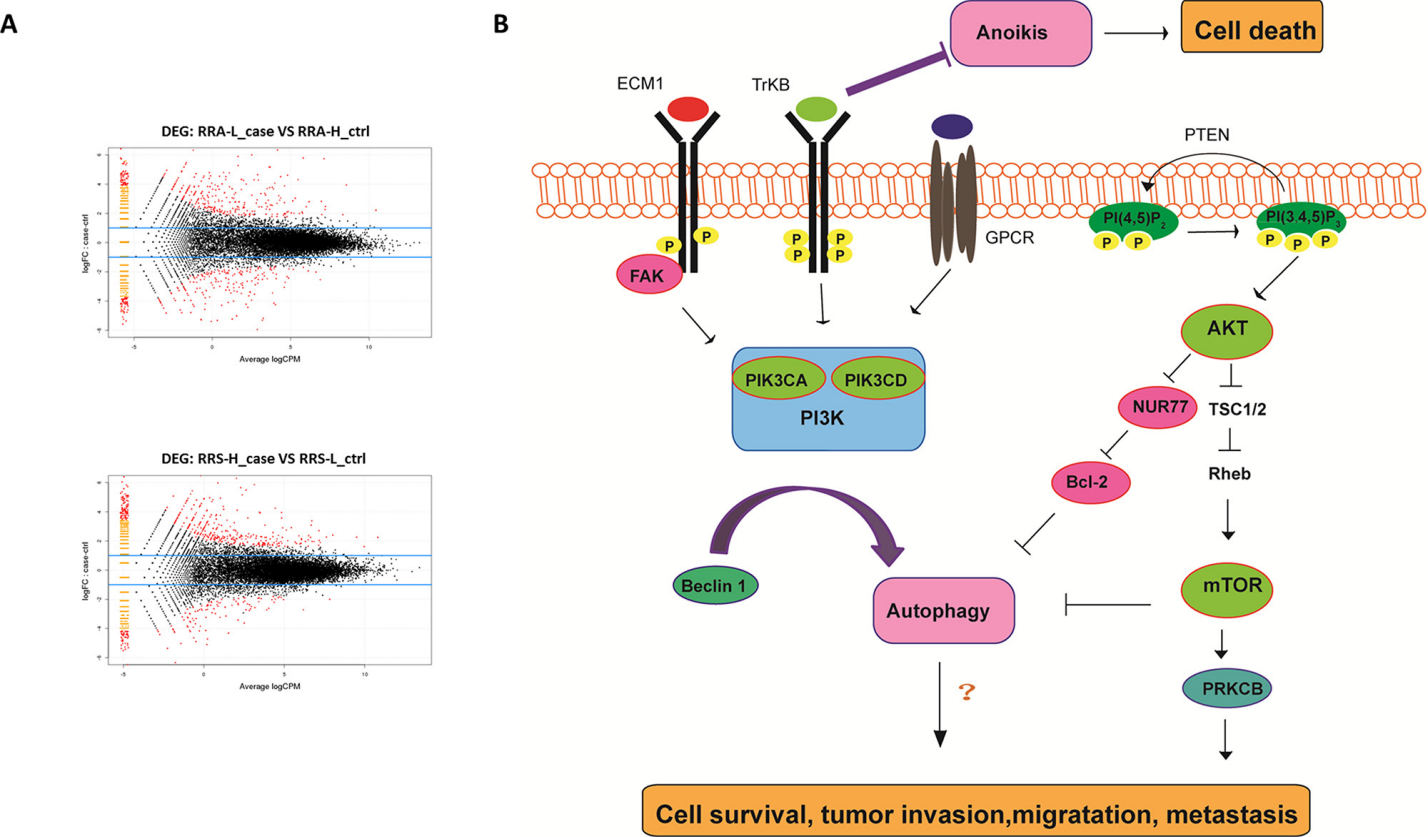
Transcriptome expression profiling of the A-H, A-L, S-H and S-L subclones **A.** RNA-Seq was conducted on the A-H, A-L, S-H and S-L cells. In total, 612 genes were significantly differentially expressed in A-H cells compared with A-L cells, and 594 genes were differentially expressed genes were identified in S-H cells compared with S-L cells (*P* < 0.05 and fold change > 2.0). **B.** The PI3K pathway plays a pivotal role in many human malignancies [34, 35]. The extracellular matrix (ECM) activates focal adhesion kinases (FAKs), and the PI3K pathway can be activated by FAKs [37]. High ECM1 expression has been correlated with poor prognosis and metastatic potential in human carcinomas [38, 39]. The most studied type I PI3Ks include the α, β, γ and δ subtypes; mutations or abnormal expression of PI3Kα(PIK3CA) are the most commonly reported in cancer [41, 42]. PI3Kγ(PIK3CD) is activated by only stimulated G-protein coupled receptor (GPCR) [43]. The survival mechanism initiated via PI3K is primarily activated through AKT. Increased AKT activation exerts anti-apoptotic effect, antagonizes cell cycle arrest, modulates angiogenesis, and activates mRNA translation via mTOR signaling [50]. P-TEN inhibits PI3K pathway activity [51, 52]. Under the regulation of AKT, Nur77 induces a conformational change in Bcl-2 and promotes apoptosis [54]. Additionally, PKC is located in downstream of mTOR. The β isoform of PKC (PRKCB) might play significant roles in promoting human breast cancers growth [55, 56].

**Figure 5:**
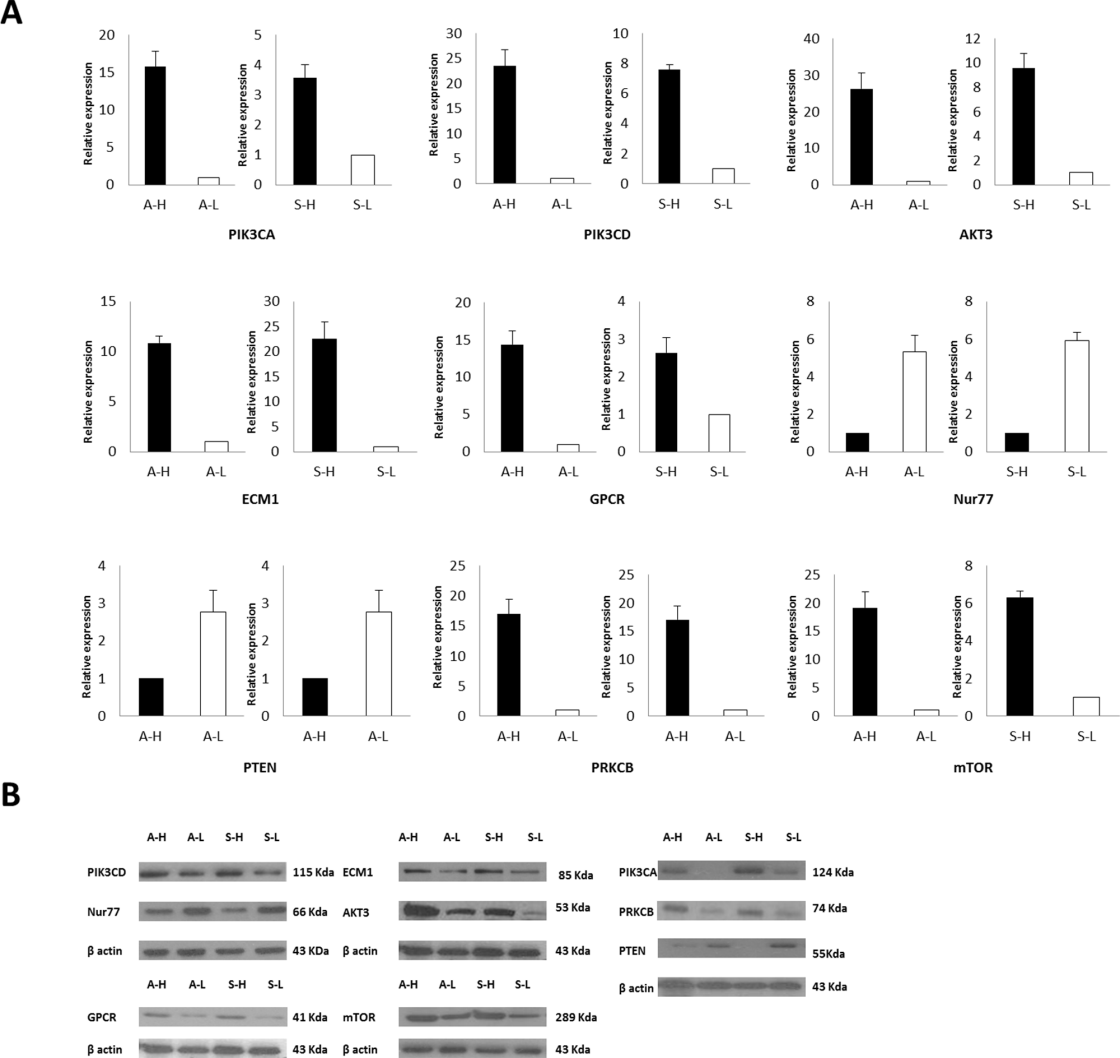
The PI3K/AKT/mTOR pathway is a potential predictor of distinct invasive and migratory capabilities in human ovarian cancer cell lines RT-PCR **A.** and Western blot analyses **B.** consistently confirmed that PIK3CA, PIK3CD, AKT3, ECM1, GPCR, mTOR and PRKCB expression was increased but that Nur77 and PTEN expression was decreased in A-H/S-H cells compared with A-L/S-L cells. PI3K/AKT/mTOR pathway Activation was associated with enhanced invasive and migratory capacities in human ovarian cancer cell lines.

RNA-Seq was conducted on the A-H, A-L, S-H and /S-L cells. In total, approximately13.0, 12.7, 10.6 and 7.9 million raw reads with a length of 75 bp were obtained for the A-H, A-L, S-H and /S-L subclones, respectively; excluding the reads lacking an anchored oligo-dT, approximately 11.0, 11.1, 8.4 and 6.3 million reads, respectively, remained that uniquely mapped to the human nuclear genome. In total, 612 genes were significantly differentially expressed in A-H cells compared with A-L cells, and 594 genes were differentially expressed genes were identified in S-H cells compared with S-L cells (*P* < 0.05 and fold-change in expression >2.0; Fig. 4A, S-Table 4, and S-Table 5). Among these cell lines, the expression of 24 genes was increased and that of 7 genes was decreased in both A-H and S-H cells compared with A-L and S-L cells, respectively. Twenty-five of the 31 genes are known to be involved in cancer. To investigate the functions of these genes in signal transduction in tumor cells, Kyoto Encyclopedia of Genes and Genomes (KEGG) pathway analysis was performed, and 5 differentially expressed genes were identified, including AKT3, ECM1, LPAR1 (also known as GPCR), NR4A1 (also known as NUR77), and PRKCB. These genes participate in the phosphatidylinositol-3-OH kinase/protein kinase B (PI3K)/AKT/mTOR pathway. These 5 differentially expressed genes, together with PIK (including the PIK3CA and PIK3CD subtypes), mTOR and PTEN, which play pivotal roles in this pathway, were selected for further verification (Fig. 4B, Table 1). RT-PCR and Western blot analyses consistently confirmed that the PIK3CA, PIK3CD, AKT3, ECM1, GPCR, mTOR and PRKCB levels were increased, whereas Nur77 and PTEN levels were decreased in A-H/S-H cells compared with A-L/S-L cells (Fig. 5).

**Table 1:** The co-existing differentially expressed genes with known roles in cancer between A-H/S-H and A-L/S-L

Gene	Gene ID	Differential expression		*P* value	
		A-H/S-H	A-L/S-L	A-H VS A-L	S-H VS S-L
**AKT3**	NM_005465.4	↑	↓	0.012	0.000
**CA12**	NM_001218	↑	↓	0.011	0.031
**COL24A1**	NM_152890	↓	↑	0.001	0.011
**CYSLTR2**	NM_020377	↑	↓	0.005	0.017
**DAPK2**	NM_014326	↑	↓	0.012	0.028
**ECM1**	NM_004425.3	↑	↓	0.004	0.048
**FAM129A**	NM_052966	↓	↑	0.001	0.014
**H19**	NR_002196	↑	↓	0.002	0.048
**HAS3**	NM_001199280	↑	↓	0.038	0.027
**HLF**	NM_002126	↑	↓	0.021	0.012
**IGFBP3**	NM_000598	↑	↓	0.013	0.000
**GPCR**	NM_0117195	↑	↓	0.047	0.013
**NUR77**	NM_001202233	↑	↓	0.005	0.015
**NRTN**	NM_004558	↑	↓	0.015	0.027
**PIK3IP1**	NM_001135911	↑	↓	0.000	0.021
**PLXDC2**	NM_032812.8	↓	↑	0.000	0.000
**PRKCB**	NM_002738.6	↑	↓	0.023	0.002
**RAB40AL**	NM_001031834	↓	↑	0.009	0.042
**RASSF5**	NM_182663	↑	↓	0.046	0.048
**RGCC**	NM_014059	↑	↓	0.048	0.001
**RYR2**	NM_001035	↑	↓	0.000	0.000
**S1PR3**	NM_005226	↓	↑	0.004	0.014
**SLC8A1**	NM_001112800	↑	↓	0.049	0.036
**TJP3**	NM_001267560	↑	↓	0.007	0.000
**WNT11**	NM_004626	↑	↓	0.002	0.048

**Note:** ↑ increase; ↓ decrease;

## DISCUSSION

Accumulating evidence in the literature [16, 17] and from our previous studies [18, 19] has demonstrated that tumor heterogeneity exists in EOC. In this study, using limiting dilution methodology and routine culture conditions, 45 subclones were isolated and established from the A2780 cell line, and 40 subclones were acquired from the SKOV3 cell line. A-H/S-H and A-L/S-L cells were identified as exhibiting the highest and lowest invasive and migratory capacities, respectively. The existence of heterogeneous subpopulations within one tumor cell line was validated.

Tumor cells must acquire the migration ability to invade tissues or vessels. Human tumors are heterogeneous; a selective advantage of the collective invasion of tumor cells could be that cell clones with different properties such as active proliferative, migratory and survival abilities could collaborate and support each other to metastasize successfully [20]. The present study demonstrated that subclones displaying high invasive and migratory capacities exhibited more aggressive phenotypes. A-H and S-H cells proliferated significantly faster, had a shorter doubling time, and included a larger proportion of cells in S phase compared with their corresponding counterparts. The apoptotic rates of A-H and S-H cells were notably lower than those of A-L and S-L cells. At both the mRNA and protein levels, reduced expression of caspase-3 and caspase-7 (pro-apoptotic factors [21]) and increased expression of Bcl-2 (an anti-apoptotic factor [22]) were observed in A-H/S-H cells. Overall, A-H/S-H cells exhibited enhanced proliferative and anti-apoptotic activities.

Anoikis can hamper metastasis by inducing apoptosis when tumor cells enter foreign environments. Anoikis tolerance is a prerequisite for tumor metastasis [23]. TrkB, a neurotrophic tyrosine kinase receptor, is a potent and specific suppressor of anoikis that activates the PI3K/PKB pathway [24, 25]. TrkB is frequently over-expressed in ovarian cancer and in other human cancers, particularly those with aggressive behavior and a poor prognosis [23, 26]. Our data showed that TrkB was over-expressed in the subclones exhibiting high invasive and migratory capacities. These findings illustrate the feasibility of using anoikis suppression as a functional property to identify metastasis-associated oncogenes and highlight its potential use in high-throughput screens for TrkB-inhibitory cancer therapeutics [25].

Autophagy is an evolutionarily conserved protein degradation pathway in eukaryotes and is essential for cell survival under nutrient-limited conditions [27]. The exact role of autophagy in cancer is complex and paradoxical, varying in distinct tumor types and stages, as well as in separate regions of the same tumor [28, 29]. Beclin1 recruits proteins from the cytosol for autophagic degradation and supplies the autophagic pathway with membrane components. Beclin 1 expression is directly correlates with autophagosome formation [30]. In addition to interacting with PI3K, Beclin 1 binds to the Bcl-2 family members Bcl-2 and Bcl-xL [31]. Thus, autophagy is often activated together with apoptosis in response to stress. Beclin1 is monoallelically deleted in human ovarian, breast and prostate cancers [32, 33]. In the present study, Beclin1 expression was decreased in subclones exhibiting high invasive/migratory capability. The synergy between autophagic defects and altered apoptotic activity might facilitate malignant differentiation and a more aggressive EOC phenotype.

In addition to enhanced proliferative, anti-apoptotic and anti-anoikis activities and suppressed autophagic activity, A-H/S-H cells exhibited significant resistance to cisplatin and taxol *in vitro* and a higher capacity for tumor formation *in vivo* compared with A-L/S-L cells. The subclones displaying high invasive and migratory capacities showed significantly greater anchorage-independent growth viability.

### The PI3K/AKT/mTOR pathway is a potential predictor of invasive/migratory capacity in human ovarian cancer cell lines

In the two pairs of targeted subclones, 5 of the differentially expressed genes revealed via RNA-Seq, i.e., ECM1, GPCR, NUR 77, AKT (AKT3) and PKC (PRKCB), are PI3K/AKT/mTOR pathway components. The PI3K pathway is involved in stimulating cell proliferation and migration and in inhibiting apoptosis; additionally, this pathway plays a pivotal role in many human malignancies [34–36]. The extracellular matrix (ECM), a series of glycosylated proteins that are secreted extracellularly, activates focal adhesion kinases (FAKs), and FAK activity is primarily are mediated by integrins. The PI3K pathway can be activated by FAKs [37]. High ECM1 expression has been correlated with poor prognosis and metastatic potential in human carcinomas [38, 39]. PI3K is the central link in the PI3K/AKT pathway; therefore, PI3K plays a crucial role in this pathway. The most studied type I PI3Ks include the α, β, γ and δ subtypes [40]; mutations or abnormal expression of PI3Kα (PIK3CA) are most commonly reported in cancer [41, 42]. PI3Kγ (PIK3CD) is activated by only stimulated G-protein coupled receptors (GPCRs) [43], and its role in cancer is controversial [44, 45]. Xie et al. [46] demonstrated that increased PI3Kγ activity propagates the metastatic signal initiated by GPCRs in breast cancer cells.

The survival mechanism initiated via PI3K is primarily activated through AKT. Increased AKT activation has been noted observed in high-grade and late-stage serous ovarian cancer [47–49], in which AKT exerts anti-apoptotic effects, antagonizes cell cycle arrest, modulates angiogenesis, and activates mRNA translation via mTOR signaling [50]. P-TEN, a candidate tumor-suppressor gene, inhibits PI3K pathway activity [51, 52]. An activating PIK3CA mutation coupled with PTEN loss is sufficient to initiate ovarian tumorigenesis in mice [53]. Under the regulation of AKT, the orphan nuclear receptor Nur77 induces a conformational change in Bcl-2 and promotes apoptosis [54]. Additionally, PKC is located downstream of mTOR. The β isoforms of PKC (PRKCB) might play significant roles in promoting human breast cancer growth [55, 56].

Briefly, the 5 differentially expressed genes that were identified by RNA-Seq, as well as PIK (PIK3CA and PIK3CD subtypes), mTOR and PTEN, which function in the PI3K/AKT/mTOR pathway, were selected for further validation of the RNA-Seq results. RT-PCR and Western blot analyses consistently confirmed that PIK3CA, PIK3CD, AKT3, ECM1, GPCR, mTOR and PRKCB expression was increased but that Nur77 and PTEN expression was decreased in A-H/S-H cells compared with A-L/S-L cells. PI3K/AKT/mTOR pathway activation was associated with enhanced invasive and migratory capacities in human ovarian cancer cell lines.

## CONCLUSIONS

Heterogeneous cell subpopulations exhibiting distinct invasive and migratory capacities co-exist within human ovarian cancer cell lines. The subclones displaying higher invasive and migratory capacities exhibit significantly increased independent viability, which might play an important role in tumor metastasis and chemotherapeutic resistance. PI3K/AKT/mTOR pathway activation is associated with higher invasive and migratory capacities in subpopulations of human ovarian cancer cell lines. Inhibiting this pathway may be useful for the chemoprevention or treatment of EOC.

## SUPPLEMENTARY TABLES






