# Supplementary material for: The PI3K/AKT/mTOR pathway is a potential predictor of distinct invasive and migratory capacities in human ovarian cancer cell lines
Source: Oncotarget. 2015 Jul 14;6(28):25520–32. doi: 10.18632/oncotarget.4550 (PMC4694849; doi:10.18632/oncotarget.4550)
Supplement: Supplementary file 1 [file oncotarget-06-25520-s001.pdf]

SUPPLEMENTARY TABLES

S-Table 1. The main materials used in this work

Materials	Supplier
Human ovarian cancer cell lines	
A2780	Cell Support Center of Institute of Basic Medical Sciences, Chinese Academy of Medical Sciences.
SKOV3	
Cell culture	
RPMI-1640	HyClone Laboratories, Inc.
Fetal bovine serum (FBS)	
Trypsin-EDTA	
Penicillin streptomycin	
A total of 96-, 48-, 24-, 12- and 6-well plates, as well as culture dishes	Corning Inc, USA
Matrigel and Boyden chambers	BD Biosciences, USA
3-(4, 5-Dimethyl-2-thiazolyl)-2, 5-diphenyl-2-H-tetrazolium bromide (MTT,)	AmrescoInc, USA
Dimethyl sulfoxide (DMSO)	AmrescoInc, USA
Propidium iodide (PI)	Sigma, USA
RNase A	Tiangen, China
Triton X-100	Sigma, USA
Annexin V-FITC/PI Apoptosis Detection Kit	BD Biosciences, USA
Trizol Reagent	Invitrogen, USA
Quantscript Reverse Transcription Kit	Tiangen, China
Radio-immune precipitation assay (RIPA) buffer	Applygen technology, China
RNA Seq	
Super Enhanced Chemoluminescence Detection Kit	Applygen Technology, China
RNeasy Mini RNA purification Kit	Qiagen, USA
TruSeq RNA library preparation kit	Illumina, USA
Qiagen gel extraction Kit	Qiagen, USA
Female BALB/c nude mice	Institute of Laboratory Animals of the Chinese Academy of Medical Sciences and maintained under pathogen-free conditions (CAMS &PUMC)*
Instruments	
ELISA reader	Multiskan MS, Labsystem, Finland
Flow cytometer	Beckman Coulter, USA
an Applied Biosystems 7500 Real-time PCR system	Applied Biosystems, USA

Note: * The institute is certified by the Association for Assessment and Accreditation of Laboratory Animal Care (AAALAC, USA).

S-Table 2. Primers for real-time PCR

Gene Name	Primer Sequence		Length (bp)
	Forward Primer	Reverse Primer	
**CASPASE 3**	AGCCCATTTCTCCATACG	TTATTGCCTCACCACCTTTAG	125
**CASPASE 7**	CTGCCTAGTGGGAGTTAGGA	GGATAGGTGAGACCAAGGTA	123
**BCL-2**	CTAAGGGTATGAAGGACCTGTA	CTCTGGAATCTAAAGGTCGT	111
**GAPDH**	TGTTGCCATCAATGACCCCTT	CTCCACGACGTACTCAGCG	201
**Trk B**	GCTTAGCTGACTTGACTCC	CTGGCATGGTTTGCAGGTA	201
**GAPDH**	TGTTGCCATCAATGACCCCTT	CTCCACGACGTACTCAGCG	102
**BECLIN 1**	CAACAAGTTTGACCATGCAAT	CTCCACATCCATCCTGTAG	112
**GAPDH**	TGTTGCCATCAATGACCCCTT	CTCCACGACGTACTCAGCG	201
**PIK3CA**	GGAGCCCAAGAATGCACAAA	TTTGTTGTCCAGCCACCATG	263
**PIK3CD**	AAATTTGAACGGTTCCGGGG	CCTCCTCTGTTTTCCCCAGT	175
**AKT3**	CATTGCTTTCAGGGCTCTTGA	AAATTGAGGGAAATGCGGCC	290
**ECM1**	TACTGTGACCGGGAGTATGC	GGGCAGTCATGTTGTGGATC	238
**LPAR1**	TCTGCTGGACTCCTGGATTG	AGATGGTGTGGTTGAGGGAG	252
**NR4A1**	AGAAGATCCCTGGCTTTGCT	CAGGGACATCGACAAGCAAG	240
**PTEN**	ACTATTCCCAGTCAGAGGCG	GAACTTGTCTTCCCGTCGTG	216
**PRKCB**	ACTGTCACCTACCCCAAGTC	CCGCGTGAAGAACTTGTCAA	237
**mTOR**	CCCATGAAAACCTCTGCCAG	TGTTTCACTGTCCTGGGAAC	282
**ACTB**	CATCCGCAAAGACCTGTACG	CCTGCTTGCTGATCCACATC	218

Note: LPAR1, namely GPCR; NR4A1, namely NUR77

S-Table 3. Details of primary and secondary antibodies used for western blotting

Antibodies	Catalog No.	Supplier
Rabbit anti-human CASPASE 3	196771-ap	Proteintech Inc (USA)
Rabbit anti-human CASPASE 7	Ab32522	ABCAM, Inc. UK
Rabbit anti-human BCL-2	12789-1-ap	Proteintech Inc (USA)
Rabbit anti-human Trk B	13129-1-ap	Proteintech Inc (USA)
Rabbit anti-human BECLIN 1	11306-1-ap	Proteintech Inc (USA)
Goat anti-human PIK3CA	sc-1332	OriGene Tech. Inc. USA
Mouse anti-human PIK3CD	ta801924	OriGene Tech. Inc. USA
Mouse anti-human AKT3	ta500001s	OriGene Tech. Inc. USA
Mouse anti-human ECM1	ab17377	ABCAM, Inc. UK
Mouse anti-human GRCP	ab119425	ABCAM, Inc. UK
Rabbit anti-human Nur77	BS3260	Biogot Tech. Inc. China
Mouse anti-human PTEN	ab79156	ABCAM, Inc. UK
Mouse anti-human (PRKCB)	ab31	ABCAM, Inc. UK
Mouse anti-human mTOR	ab87540	ABCAM, Inc. UK

S-Table 4. Differentially-expressed genes_A-H_VS_A-L

S-Table 5. Differentially-expressed genes_S-H_VS_S-L
